# Pharmacokinetic modeling of P-glycoprotein function at the rat and human blood–brain barriers studied with (*R*)-[^11^C]verapamil positron emission tomography

**DOI:** 10.1186/2191-219X-2-58

**Published:** 2012-10-16

**Authors:** Julia Müllauer, Claudia Kuntner, Martin Bauer, Jens P Bankstahl, Markus Müller, Rob A Voskuyl, Oliver Langer, Stina Syvänen

**Affiliations:** 1 Biomedical Systems, Health & Environment Department, AIT Austrian Institute of Technology GmbH, Seibersdorf, 2444, Austria; 2 Department of Clinical Pharmacology, Medical University of Vienna, Währinger Gürtel 18-20, Vienna, 1090, Austria; 3 Department of Nuclear Medicine, Hannover Medical School, Carl-Neuberg-Straße 1, Hannover, 30625, Germany; 4 Epilepsy Institutes of The Netherlands Foundation, Achterweg 5, Heemstede, 2103 SW, The Netherlands; 5 Division of Pharmacology, Leiden University, Einsteinweg 55, Leiden, 2333 CC, The Netherlands; 6 Department of Public Health and Caring Sciences, Uppsala University, Rudbecklaboratoriet, Uppsala, 751 85, Sweden

**Keywords:** Nonlinear mixed effects modeling, Positron emission tomography, (*R*)-[^11^C]verapamil, P-glycoprotein, Tariquidar, Pilocarpine-induced epilepsy, Species differences

## Abstract

**Background:**

This study investigated the influence of P-glycoprotein (P-gp) inhibitor tariquidar on the pharmacokinetics of P-gp substrate radiotracer (*R*)-[^11^C]verapamil in plasma and brain of rats and humans by means of positron emission tomography (PET).

**Methods:**

Data obtained from a preclinical and clinical study, in which paired (*R*)-[^11^C]verapamil PET scans were performed before, during, and after tariquidar administration, were analyzed using nonlinear mixed effects (NLME) modeling. Administration of tariquidar was included as a covariate on the influx and efflux parameters (*Q*_in_ and *Q*_out_) in order to investigate if tariquidar increased influx or decreased outflux of radiotracer across the blood–brain barrier (BBB). Additionally, the influence of pilocarpine-induced status epilepticus (SE) was tested on all model parameters, and the brain-to-plasma partition coefficient (*V*_T-NLME_) was calculated.

**Results:**

Our model indicated that tariquidar enhances brain uptake of (*R*)-[^11^C]verapamil by decreasing *Q*_out_. The reduction in *Q*_out_ in rats during and immediately after tariquidar administration (sevenfold) was more pronounced than in the second PET scan acquired 2 h after tariquidar administration (fivefold). The effect of tariquidar on *Q*_out_ in humans was apparent during and immediately after tariquidar administration (twofold reduction in *Q*_out_) but was negligible in the second PET scan. SE was found to influence the pharmacological volume of distribution of the central brain compartment *V*_br1_. Tariquidar treatment lead to an increase in *V*_T-NLME_, and pilocarpine-induced SE lead to increased (*R*)-[^11^C]verapamil distribution to the peripheral brain compartment.

**Conclusions:**

Using NLME modeling, we were able to provide mechanistic insight into the effects of tariquidar and SE on (*R*)-[^11^C]verapamil transport across the BBB in control and 48 h post SE rats as well as in humans.

## Background

About one-third of patients with epilepsy are pharmacoresistant and do not respond adequately to antiepileptic drug therapy [1]. The blood–brain barrier (BBB) has a major role in regulating the transport of antiepileptic drugs to their target site of action. Drug penetration across the BBB is influenced by several mechanisms, such as passive diffusion, active influx, and active efflux. Regional overactivity of efflux transporters at the BBB is thought to contribute to drug resistance by impeding therapeutically effective concentrations of antiepileptic drugs at their sites of action [2]. P-glycoprotein (P-gp), which is physiologically located at the luminal membrane of brain capillary endothelial cells, is currently one of the most widely studied efflux transporters at the BBB. Positron emission tomography (PET) with carbon-11-labeled P-gp substrates, such as (*R*)-^11^C]verapamil or ^11^C]-*N*-desmethyl-loperamide, has been evaluated as a tool for *in vivo* imaging of P-gp function in different species [3-6]. However, these radiotracers are high-affinity P-gp substrates and consequently display very low brain concentrations, limiting their suitability as PET tracers [5,7,8]. This drawback can be overcome by modulation of P-gp with the third-generation P-gp inhibitor tariquidar, which leads to increased brain uptake of these radiotracers. After partial inhibition of P-gp with 3 mg/kg tariquidar, regional differences in P-gp expression and functionality between naïve and status epilepticus (SE) rats become evident [9,10]. Additionally, P-gp at the BBB has been found to be upregulated after acute seizure activity like SE or in chronic epilepsy [11-15]. Further, species-dependent differences in P-gp expression and functionality at the BBB have been described and discussed in literature [16-18], including the P-gp mediated interaction between (*R*)-^11^C]verapamil and tariquidar at the human and rat BBB studied by Bauer et al. [18]. Despite several published studies, there is still an ongoing debate, whether P-gp inhibition with tariquidar or other inhibitors is enhancing the brain uptake of substrate radiotracers by increasing the influx or decreasing outflux across the BBB of the radiotracer [9].

In PET research, pharmacokinetic (PK) modeling (compartment modeling) is used for detailed quantitative analysis of PET data. Each individual is analyzed separately, and group averages and variability are subsequently based on the individual estimates. An alternative way to analyze PK-pharmacodynamic (PD) data is nonlinear mixed effects (NLME) modeling, often referred to as population modeling. This modeling approach is routinely used in pharmaceutical research and has also found to be suitable for PET research [19-29]. A population approach analyzes data from all subjects simultaneously and gives a description of the PK in the typical subject as well as the variation in the study population. Syvänen et al. [26] analyzed data from a (*R*)-^11^C]verapamil PET study in naïve and post SE rats (seven days after kainate treatment) with both a PET PK-modeling approach and a NLME-modeling approach and concluded that both approaches produced similar PK parameter estimates, but that NLME modeling provided more precise parameter estimates.

**Figure 1:**
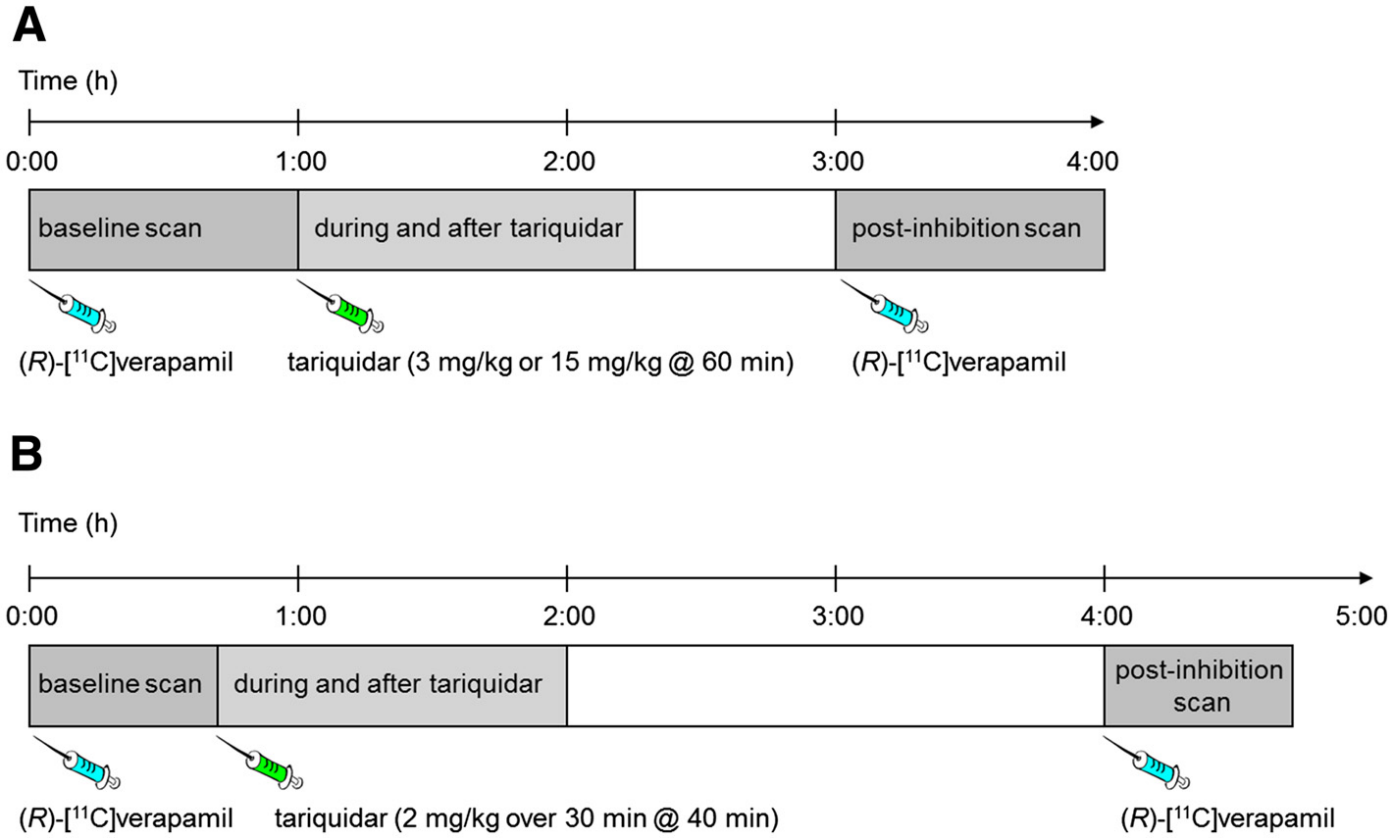
Scheme of the setups for the preclinical study (A) and the clinical study (B).

In the present study, we analyzed data obtained from a preclinical PET study in naïve rats and rats at 48 h after pilocarpine-induced SE [9] and a clinical study in healthy volunteers [30]. In both studies, subjects underwent paired (*R*)-^11^C]verapamil PET scans before, during, and after administration of tariquidar (rats, 3 and 15 mg/kg; humans, 2 mg/kg) (see Figure 1). Tariquidar was administered during the first PET scan and PET data acquisition continued during and after tariquidar administration. Both in rats and in humans a pronounced and immediate increase in (*R*)-^11^C]verapamil brain uptake during treatment with tariquidar was observed. In the original analysis of the rat data [9], (*R*)-^11^C]verapamil brain curves obtained during and after tariquidar treatment in the first PET scan (60 to 140 min) were not included in PET PK modeling as standard PET approaches do not handle this type of data. In the original analysis of the human data [30], however, efforts were made to analyze the time course of the effect of tariquidar administration on activity in brain during the first PET scan using an indirect response PK-PD model proposed by Syvänen et al. [31] (see supplemental data of Wagner et al. [30]). These results suggested that the (*R*)-^11^C]verapamil brain curves obtained during and immediately following tariquidar treatment may contain important information that may contribute to the model structure and parameter outcome. Therefore, we hypothesized that the use of NLME modeling may provide mechanistic information of the PK of (*R*)-^11^C]verapamil in plasma and brain including quantification of the influence of tariquidar-induced P-gp inhibition and pilocarpine-induced SE on (*R*)-^11^C]verapamil brain and plasma PK. The specific aims of this study were to investigate whether the observed increase in (*R*)-^11^C]verapamil brain uptake during and immediately after tariquidar treatment influences PK model parameter estimates and if regional differences in certain brain regions as recently discussed by Bankstahl et al. [9] become more pronounced when these data are included in the analysis. Additionally, it was studied whether the enhancement of brain uptake of (*R*)-^11^C]verapamil after tariquidar administration was caused by increased influx or decreased outflux of the radiotracer. Finally, the developed model based on the rat dataset was applied to the human data set to identify potential species differences in P-gp function.

## Methods

### Preclinical data set

The preclinical dataset was recently published by Bankstahl et al. [9], and the study setup is illustrated in Figure 1A. The study was approved by the Institutional Animal Care and Use Committee, and all study procedures were performed in accordance with the European Communities Council Directive of November 24, 1986 (86/609/EEC). All efforts were made to minimize both the suffering and the number of animals used in this study.

Naïve control (*n* = 11) and 48-h post SE (*n* = 10) female Sprague–Dawley rats (Harlan Nederland, Horst, Netherlands) weighing 260±28 g underwent paired (*R*)-^11^C]verapamil PET scans. The number of animals, animal weights, injected doses, and injection times of all treatment groups are summarized in Table 1. All rats underwent a 140-min dynamic baseline PET scan starting simultaneously with (*R*)-^11^C]verapamil administration given as an intravenous bolus (I.V.) on a microPET Focus 220 scanner (Medical Solutions, Siemens Knoxville, TN, USA). Tariquidar (3 or 15 mg/kg) was administered at 60 min after the start of the baseline scan as an I.V. bolus over 60 sec. A second 60-min dynamic (*R*)-^11^C]verapamil PET scan, referred to as post-inhibition scan, was started 2 h after tariquidar administration. In parallel to the measurement of (*R*)-^11^C]verapamil brain concentrations with PET, (*R*)-^11^C]verapamil concentration in blood and plasma (mean plasma-to-blood ratio of (*R*)-^11^C]verapamil = 1.29±0.10 [32]) was obtained with continuous arterial blood sampling. In addition, (*R*)-^11^C]verapamil concentration in plasma was corrected for radiolabelled metabolites. More information on the PET scan procedure, the study setup, the arterial blood sampling, and the metabolite correction can be found in the previously published studies by Bankstahl et al. [9] and Kuntner et al. [10]. For modeling, (*R*)-^11^C]verapamil concentration-time curves before and after tariquidar administration, expressed in units of kilobecquerels per milliliters (kBq/ml), were extracted from eight brain regions of interest (whole brain (WB), corpus striatum (CS), entorhinal cortex (EC), septal hippocampus (Shipp), temporal hippocampus (THipp), thalamus (Th), cerebellum (Cer), and frontal motor cortex (FMC)) as described previously [10]. For comparison with human data, activity concentrations were normalized to injected activity per body weight and expressed as standardized uptake value (SUV).

**Table 1:** **Number of animals (*n*), weight at time of PET, injected doses (average ± standard deviation) of (*R*)-[**^**11**^**C]verapamil for baseline and post-inhibition scans in naïve and 48-h post SE rats and different treatments with 3 or 15 mg/kg of tariquidar**

	**Naïve and 3 mg/kg tariquidar treated**	**Naïve and 15 mg/kg tariquidar treated**	**48-h post SE and 3 mg/kg tariquidar treated**	**48-h post SE and 15 mg/kg tariquidar treated**	**All groups**
*N*	7	4	5	5	21
Body weight (g)	260 ± 5	307 ± 18	234 ± 14	247 ± 16	260 ± 28
Baseline (*R*)-[^11^C]verapamil (MBq)	97 ± 27	88 ± 17	84 ± 20	94 ± 25	91 ± 22
I.V. injection time (sec)	18 ± 7	39 ± 10	19 ± 2	25 ± 11	24 ± 11
Post-inhibition (*R*)-[^11^C]verapamil (MBq)	93 ± 27	101 ± 28	91 ± 9	92 ± 16	94 ± 21
I.V. injection time (sec)	18 ± 6	36 ± 5	16 ± 2	32 ± 18	24 ± 13

### Clinical data set

The clinical dataset has been described in detail by Wagner et al. [30]. The study setup is illustrated in Figure 1B. The study protocol was approved by the local Ethics Committee and was performed in accordance with the Declaration of Helsinki (1964) in the revised version of 2000 (Edinburgh), the Guidelines of the International Conference of Harmonization, the Good Clinical Practice Guidelines, and the Austrian Drug Law (Arzneimittelgesetz). All subjects were given a detailed description of the study, and their written consent was obtained before they enrolled in the study.

Five healthy male volunteers with an average age of 32±8 years and an average body weight of 74±5 kg underwent paired (*R*)-[^11^C]verapamil PET scans of 120- and 40-min duration, respectively, with an interval of 2 h between the two scans on an Advance PET scanner (GE Medical Systems, Waukesha, WI, USA). Dynamic PET and arterial blood sampling were started at the time of radiotracer injection. Forty minutes after the start of the baseline scan, tariquidar was administered at a dose of 2 mg/kg of body weight I.V. over 30 min. Post-inhibition scan was performed at 2 h and 50 min after the end of tariquidar infusion. Number of participants, body weight, injected doses, and radiotracer injection times are summarized in Table 2.

**Table 2:** **Number of human volunteers (*n*), weight at time of PET, injected doses (average ± standard deviation) of (*R*)-[**^**11**^**C]verapamil for baseline, and post-inhibition scans after tariquidar (2 mg/kg) treatment**

	**Healthy volunteers and 2 mg/kg tariquidar treated**
Number	5
Body weight (kg)	74±5
Baseline (*R*)-[^11^C]verapamil (MBq)	379±11
I.V. injection time (sec)	51±10
Post-inhibition (*R*)-[^11^C]verapamil (MBq)	389±15
I.V. injection time (sec)	43±11

For modeling, (*R*)-^11^C]verapamil concentration-time curves before and after tariquidar administration, expressed in kBq/ml, were extracted from a WB gray matter region as described previously [30].

### PK modeling: PET approach

PET PK modeling parameters for rats and human were taken from Bankstahl et al. [9] and Wagner et al. [30], respectively. Individual profiles from each animal or human were analyzed using the common data analysis approaches for PET (PK modeling of individual profiles) [33,34]. In both studies, a two-tissue-4-rate constant (2T4K) compartment model was applied to estimate the brain-to-plasma partition coefficient, referred to as the volume of distribution in PET literature (*V*_T-2T4K_), and the rate constants describing exchange of radioactivity between the plasma and the two brain tissue compartments. To further obtain a model-independent estimate of the brain-to-plasma partition coefficient, Logan graphical analysis [34] was used (*V*_T-Logan_). Data obtained during and immediately after tariquidar treatment, i.e., the last part of the baseline scans, were not included when estimating *V*_T*-*2T4K_ and *V*_T*-*Logan_.

### NLME modeling

For modeling of the metabolite-corrected plasma and brain concentration-time curves during the entire scan period, NLME modeling in NONMEM VI (GloboMax LLC, Hanover, MD, USA) was applied in order to predict the rate constants describing the PK of (*R*)-^11^C]verapamil and the effects of tariquidar on (*R*)-^11^C]verapamil PK. Data from all subjects and all scans were analyzed simultaneously to yield population estimates of PK parameters as well as estimates of inter-animal variability. Covariate analysis allowed for the identification of the specific sources of variability. The subroutine ADVAN 9 and first-order conditional estimation with interaction were used throughout the modeling procedure. Model selection was based on the objective function value (OFV, the lowest value corresponds to the best model), model parameter uncertainty, and graphic analysis using Xpose 4 [35] implemented in software R 2.7.1. (The R Foundation for Statistical Computing). Outcome parameters were viewed and compared in Census [36]. For nested models, OFV reductions of 3.83, 6.63, and 10.83 units correspond to improved fits at *p* < 0.05, *p* < 0.01, and *p* < 0.001 levels. The inter-individual variation of the parameters was described by the exponential variance model:

(1) $$\theta_{i}=\theta_{\text{pop}}*exp\left(\eta_{i}\right)\text{,}$$

where *θ*_*i*_ is the parameter in the *i*th animal, *θ*_pop_ is the parameter in a typical animal, and *η*_*i*_ is the inter-animal variability, which is assumed to be normally distributed around zero with a standard deviation *ω*, to distinguish the *i*th animal from the typical value as predicted from the regression model. Inter-animal variation was studied on all parameters and was included if the model was improved significantly (OFV > 3.83). To test the significance of the covariate inclusion, e.g., effect of tariquidar and SE, a stepwise forward addition and backward deletion approach was applied, and covariates were only kept in the model if they significantly improved the model. Finally, proportional error models were included for the residual variability, i.e., variability that remained unexplained after inclusion of inter-animal variability and covariates. More comprehensive description of NLME modeling can be found elsewhere, for example in the paper by Pillai et al. [37].

The model development was carried out using the rat dataset (WB region) and the model was built in sequential steps. The first step was to develop a PK model for (*R*)-[^11^C]verapamil plasma concentration-time profiles. Two and three compartment models were evaluated. Treatment (tariquidar-treated or tariquidar-untreated) and rat group (control or 48-h post SE) were defined as covariates (Eff_tariquidar_ and Eff_SE_) and their effects on the parameter estimates were studied. Next, a PK model of (*R*)-[^11^C]verapamil concentration-time profiles in brain was developed. Two and three compartment models were evaluated, and again treatment and rat group were defined as covariates. Finally, in the last step of the model development, the PK models for (*R*)-[^11^C]verapamil kinetics in plasma and brain were merged.

**Figure 2:**
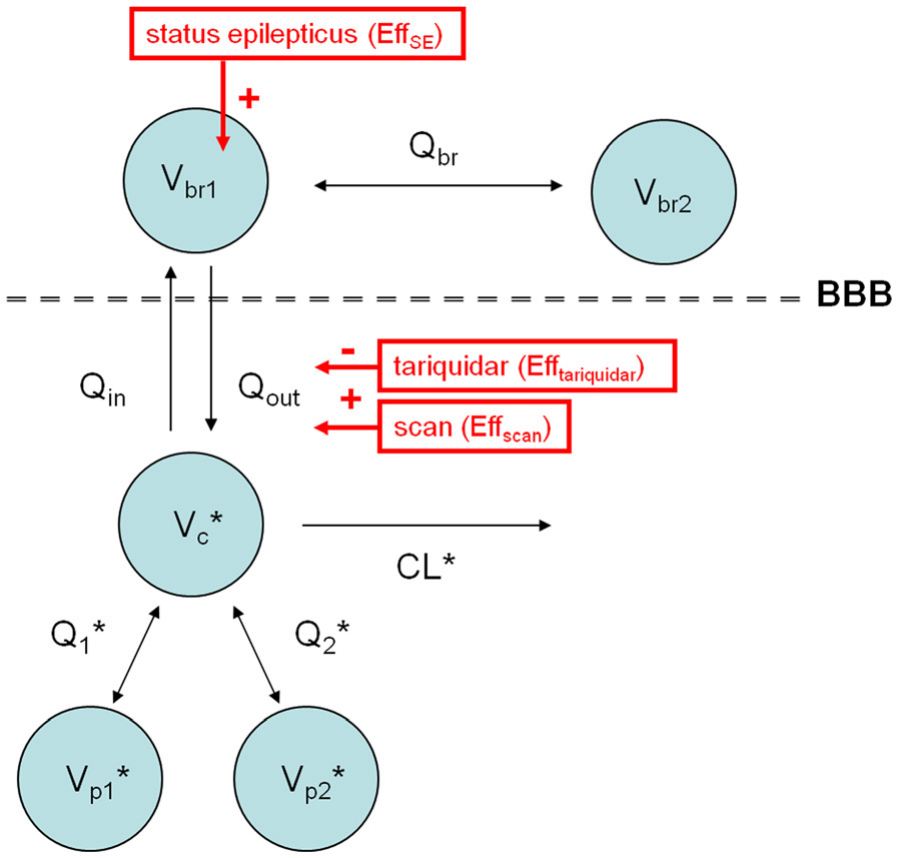
**The final population model.***V*_c_, *V*_p1_ and *V*_p2_ are the pharmacological volumes of distribution in central and two peripheral plasma compartments, while *V*_br1_ and *V*_br2_ are the volumes of distribution in central and peripheral brain compartments, respectively. CL, *Q*_1_, *Q*_2_, *Q*_in_, *Q*_out_, and *Q*_br_ are total body clearance, bidirectional clearance between plasma and peripheral compartment 1, bidirectional clearance between plasma and peripheral compartment 2, clearance into the brain, clearance out of the brain, and bidirectional clearance between central and peripheral brain compartments, respectively. Plus sign (+) indicates an increase in *V*_br1_ due to rat group (Eff_SE_) and the effect of tariquidar (Eff_tariquidar_), resulting in a decrease in *Q*_out_. Asterisk (*) indicates the parameter estimates, which were fixed during modeling procedure, though *V*_br2_ was only fixed in rats.

The increased uptake of (*R*)-^11^C]verapamil into the brain after administration of tariquidar was assumed to be either due to increased transport of (*R*)-^11^C]verapamil into the brain (i.e., increased *Q*_in_, Equation 2) or decreased transport of (*R*)-^11^C]verapamil out of the brain (i.e., decreased *Q*_out_, Equation 3) [25].

(2) $$\frac{dVER_{br1}}{dt}=D\cdot Q_{\mathrm{in}}\cdot VER_{c}-Q_{\mathrm{out}}\cdot VER_{br1}$$

(3) $$\frac{dVER_{br1}}{dt}=Q_{\mathrm{in}}\cdot VER_{c}-D\cdot Q_{\mathrm{out}}\cdot VER_{br1}$$

(4) $$\frac{dVER_{br2}}{dt}=Q_{\mathrm{br}}\cdot VER_{br1}-Q_{\mathrm{br}}\cdot VER_{br2}\text{,}$$

where VER_br1_ and VER_c_ are the (*R*)-^11^C]verapamil concentrations in the central brain compartment and in plasma, respectively, and *D* (Equations 2 and 3) describes the effect of tariquidar on the transport of (*R*)-^11^C]verapamil between plasma and brain. *D* was considered to affect the clearance out of the brain (*Q*_out_) or the clearance into the brain (*Q*_in_). *Q*_br_ is the bidirectional clearance between the central and the peripheral brain compartments (see also Figure 2). Different models for describing *D* were tested, including indirect effect models based on tariquidar plasma concentration or administered dose or as a combination of categorical covariates for tariquidar treatment and scan (see ‘Results’ section). A second (peripheral) brain compartment was evaluated for (*R*)-^11^C]verapamil, where VER_br2_ is the (*R*)-^11^C]verapamil concentration in the peripheral slow equilibrating brain compartment (Equation 4). The final PK model developed for the WB region of the rat was then applied to the selected brain regions of interest (CS, EC, Shipp, THipp, Th, Cer, and FMC), respectively. To investigate whether the increase of (*R*)-^11^C]verapamil brain uptake during and after treatment with tariquidar (60 to 140 min of baseline scan) influenced the model outcome parameters, the final model was applied to the first 60 min of the baseline (i.e., excluding the data acquired during and immediately after tariquidar administration) and the post-inhibition scan only. The effect of tariquidar (*D*) was studied on the *Q*_in_ and the *Q*_out_ of (*R*)-^11^C]verapamil brain uptake. Additionally, the model was applied to the human data set, and the influence of including or excluding (*R*)-^11^C]verapamil brain concentrations during and after the tariquidar administration (40 to 120 min of baseline scan) on the model outcome parameters was evaluated. Volume of distribution (*V*_T-NLME_) was calculated as the ratio *Q*_in_*/Q*_out_ and compared with V_T-2T4K_ values obtained with standard PET PK modeling.

### Statistics

Differences between groups were analyzed by 2-way ANOVA including Bonferroni correction using PRISM 5 software (GraphPad Software, La Jolla, CA). The level of statistical significance was set to *p* < 0.05.

## Results

**Figure 3:**
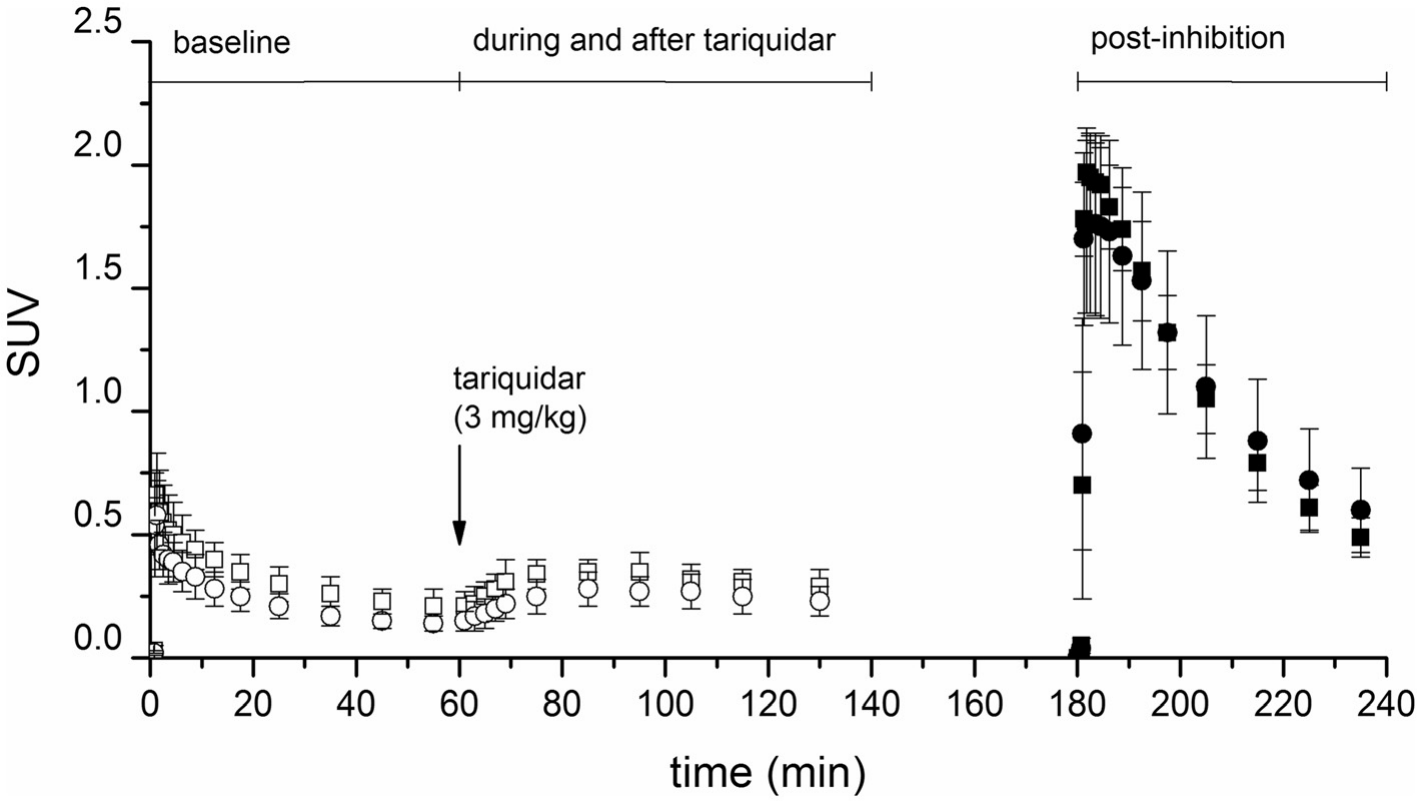
**WB (*R*)-[**^**11**^**C]verapamil time-activity curves in rats.** Average ± standard deviation (*R*)-[^11^C]verapamil concentration-time curves in rat WB expressed as SUV for naïve (squares) and 48-h post SE (circles) animals at baseline (open symbols) and at post-inhibition (full symbols). Tariquidar (3 mg/kg) was administered 60 min after the start of the baseline scan as an I.V. bolus over 60 sec (arrow).

**Figure 4:**
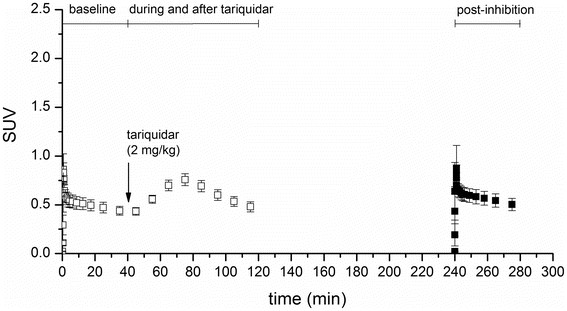
**WB (*R*)-[**^**11**^**C]verapamil time-activity curves in humans.** Average ± standard deviation (*R*)-[^11^C]verapamil concentration-time curves in human WB gray matter expressed as SUV at baseline (open symbols) and at post-inhibition (full symbols). Tariquidar (2 mg/kg) was administered 40 min after the start of the baseline scan as an infusion over 30 min (arrow).

WB (*R*)-[^11^C]verapamil time-activity curves expressed as SUV at baseline and in the post-inhibition scan are shown for naïve and 48-h post SE rats in Figure 3 and for humans in Figure 4. Both in rats and humans, tariquidar administration during the baseline scan resulted in an immediate rise in brain activity concentrations.

### NLME modeling of plasma and brain PK with and without tariquidar administration

**Figure 5:**
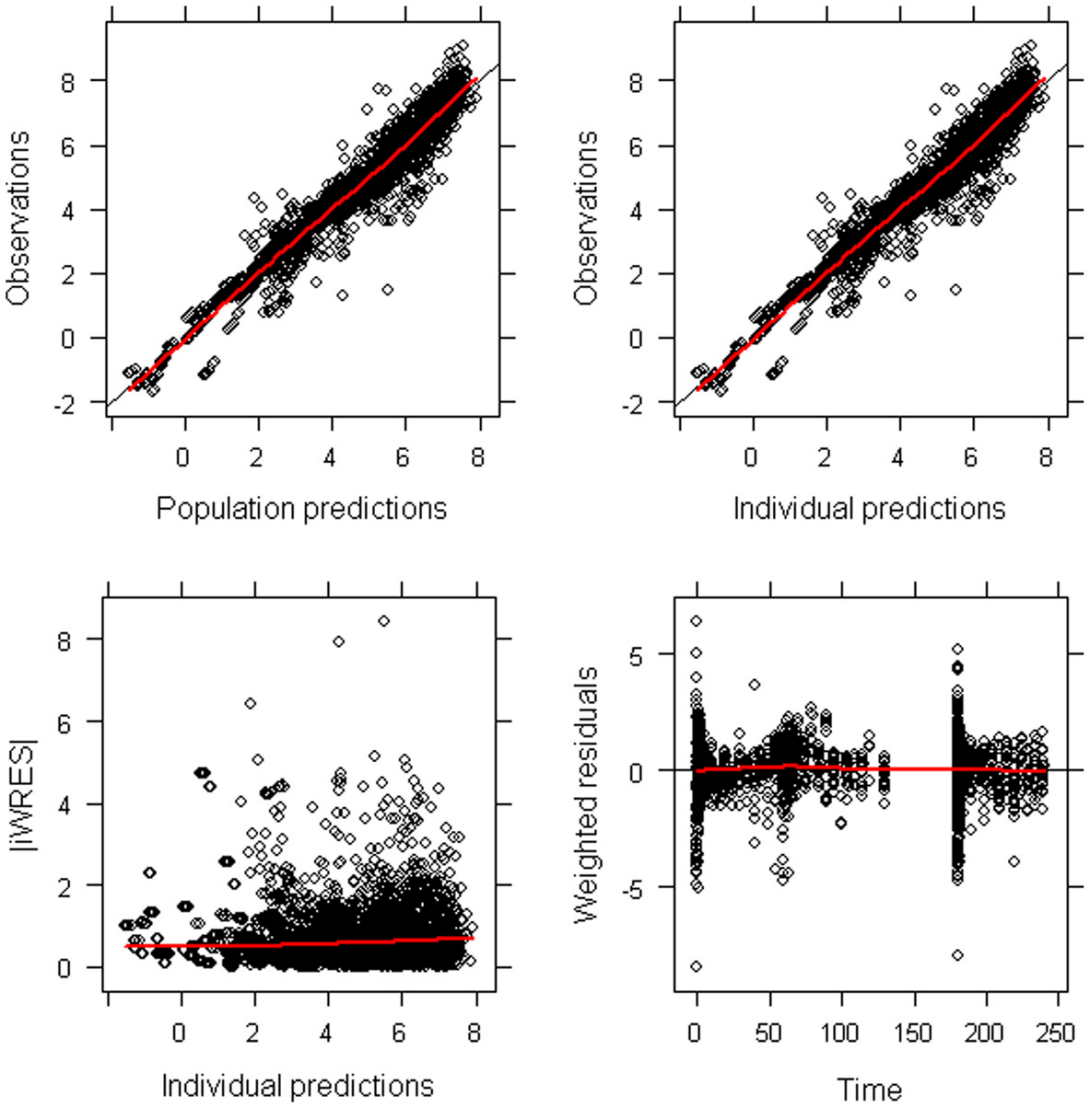
**Diagnostic plots for the final population model including both brain and plasma (*R*)-[**^**11**^**C]verapamil concentrations.** Each individual data point is represented by one dot. The red line represents the average observation. Observations are plotted against population predictions (upper left panel) and individual predictions (upper right panel). Data points are randomly distributed along the line of identity, indicating that the concentrations are adequately described by the model. Absolute individual weighted residuals versus individual predictions (lower left panel) and weighted residuals versus time (lower right panel) are plotted. Most residuals are clustered around zero, while there are outliers at early time points.

**Figure 6:**
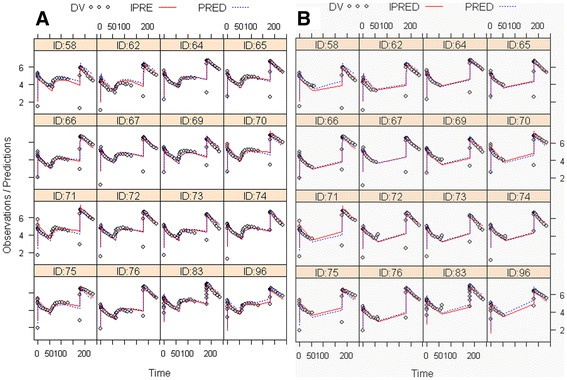
**Modeling results of (*R*)-[**^**11**^**C]verapamil concentration-time curves in WB of rats in ln(Bq/ml) over time (min).** (**A**) WB was compared with (**B**) WB* excluding the increase of (*R*)-[^11^C]verapamil brain uptake after treatment with tariquidar from 60 to 140 min. Tariquidar (3 or 15 mg/kg) was administered at 60 min after start of tracer injection as an I.V. bolus over 60 sec and the post-inhibition scan was started 2 h after tariquidar administration. Each panel represents one animal (not all 21 are shown), open gray circles represent measurements (DV), solid red lines represent the model predictions for individual rats (IPRE), and broken blue lines represent the population model predictions (PRED).

**Figure 7:**
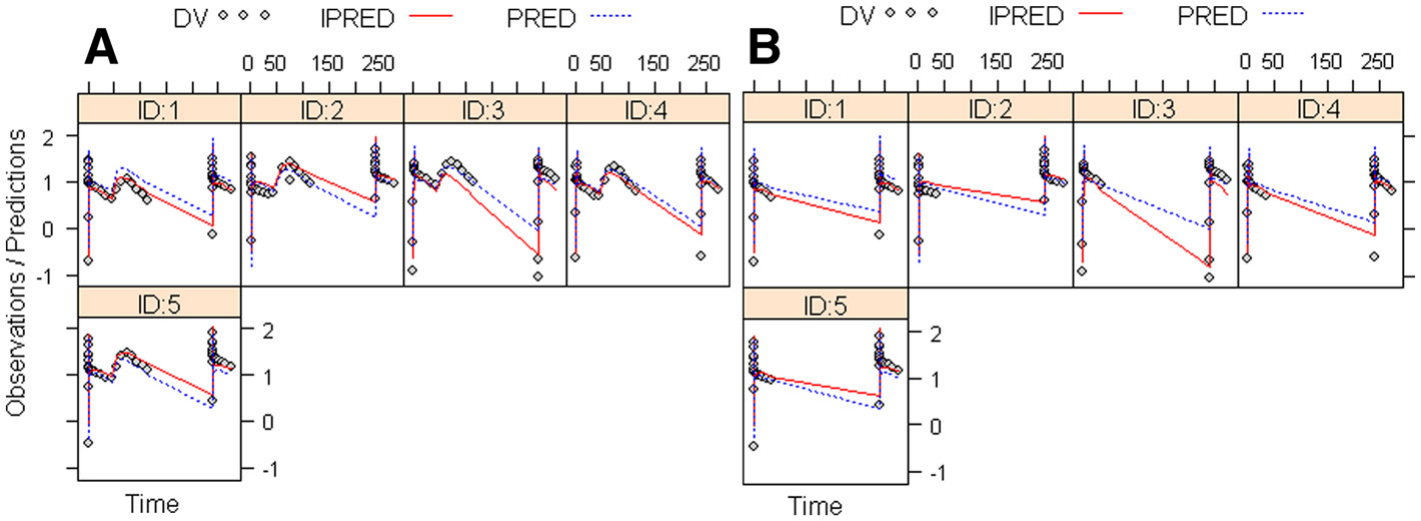
**Modeling results of (*R)*-[**^**11**^**C]verapamil concentration-time curves in WB gray matter in ln(Bq/ml) over time (min).** (**A**) WB gray matter was compared with (**B**) WB* gray matter excluding the increase of (*R*)-[^11^C]verapamil brain uptake after treatment with tariquidar. Tariquidar (2 mg/kg) was administered at 40 min after start of tracer injection as an intravenous infusion over 30 min and post-inhibition scan was started 2 h 50 min after end of tariquidar infusion. Each panel represents one volunteer, open gray circles represent measurements (DV), solid red lines represent the model predictions for each individual subject (IPRE), and broken blue lines represent the population model predictions (PRED).

NLME modeling was used to study the kinetics of (*R*)-^11^C]verapamil in plasma and brain. Metabolite corrected plasma curves were best described with a three compartment model. Inclusion of covariates for describing the effect tariquidar treatment (Eff_tariquidar_) and rat group (Eff_SE_) did not result in a significant model improvement, when applied to any of the PK parameters of the plasma model and were, therefore, not included. Parameter estimates, the relative standard error (%), and the inter-animal variability for the plasma model are given in Table 3. Brain PK in both rats and humans was best described with a two-compartment model. The brain model was combined with the plasma PK model. For rats, plasma parameter estimates were fixed according to the best plasma model (see Table 3). In addition, the pharmacological volume of distribution of the peripheral brain compartment, *V*_br2_, was fixed to a value of 2 ml, i.e. the total volume of a rat brain [38]. All other brain parameter estimates were allowed to freely change. For the human data, plasma parameters were also fixed, whereas the brain parameter estimates were allowed to freely change. The final model is shown in Figure 2, and the model diagnostics plots for the rat model are shown in Figure 5. Population parameter estimates of the (*R*)-^11^C]verapamil brain model for all studied brain regions of interest are shown in Table 4 for rats and humans. (*R*)-^11^C]verapamil concentration-time profiles in rats and model predictions of WB (data from 0 to 140 min and 180 to 240 min) and WB^*^ (data from 0 to 60 min and 180 to 240 min) are shown in Figure 6. For the human data modeling, results of (*R*)-^11^C]verapamil concentration-time curves of the WB gray matter (data from 0 to 120 min and 240 to 280 min) and WB^*^ gray matter (data from 0 to 40 min and 240 to 280 min) are shown in Figure 7. Structural model parameters were obtained for the clinical data (Table 3), but due to the relatively small sample size (*n* = 5), reliable estimates of inter-individual variation and standard deviations could not be obtained.

**Table 3:** **Population parameter estimates and relative standard errors (%) of the (*R*)-[**^**11**^**C]verapamil plasma model using mixed effects modeling of the WB region of interest of rats and humans**

**Pharmacokinetic parameters**	**Rat**		**Human estimates**
	**Estimates**	**Inter-animal variability**	
*V*c (ml)	38.8	0.162	2580
	(7.27)	(52.3)	(−)
*V*_p1_ (ml)	141	0.169	57900
	(17.6)	(23.3)	(−)
*V*_p2_ (ml)	1580	-	41100
	(17.6)		(−)
CL (ml·min^−1^)	16.4	0.162	538
	(10.3)	(52.3)	(−)
*Q*_1_ (ml·min^−1^)	29.1	0.169	6550
	(7.77)	(23.3)	(−)
*Q*_2_ (ml·min^−1^)	22.3	0.162	5880
	(10.0)	(52.3)	(−)
Residual error plasma	0.482	-	0.654
	(6.93)		(−)

For definition of parameters, please refer to the legend of Figure 2. The RSA are given in parentheses after the estimates.

**Table 4:** **Population parameter estimates and relative standard errors (%) of (*R*)-[**^**11**^**C]verapamil brain model are shown for all investigated brain regions**

	**Species**										
	**Rat**									**Human**	
**Brain region**	WB^*,a^	**WB**^**b**^	**CS**	**EC**	**SHipp**	**THipp**	**Th**	**Cer**	**FMC**	WB^*,a^	**WB**^**b**^
OFV	−604	−1676	−1420	−1652	−1500	−954	−1559	−1562	−1365	7799	−357
Model parameters											
*V*_br1_ (ml)	0.132	0.132	0.119	0.155	0.151	0.129	0.156	0.192	0.088	2.29	3.17
	(10.5)	(11.3)	(10)	(11.4)	(9.74)	(27.2)	(12.2)	(19.2)	(15.8)	(−)	(−)
*V*_br2_ (ml)	2	2	2	2	2	2	2	2	2	13.0	18.4
	(−)	(−)	(−)	(−)	(−)	(−)	(−)	(−)	(−)	(−)	(−)
*Q*_in_ (ml·min^−1^)	5.48	5.62	2.05	3.32	2.49	1.98	2.43	2.44	1.79	713	588
	(13.0)	(12.5)	(12.8)	(16.8)	(12.7)	(24.5)	(11.3)	(14.2)	(19.2)	(−)	(−)
*Q*_out_ (ml·min^−1^)	3.56	3.60	3.45	3.91	4.56	2.99	4.39	3.01	1.97	1410	1080
	(12.6)	(12.5)	(12.5)	(16.3)	(12.5)	(21.8)	(11.3)	(13.9)	(19.1)	(−)	(−)
*Q*_br_ (ml·min^−1^)	0.115	0.115	0.114	0.111	0.113	0.108	0.129	0.118	0.12	1.26	1.71
	(3.2)	(3.05)	(3.43)	(3.71)	(3.87)	(4.63)	(3.45)	(3.24)	(3.58)	(−)	(−)
Residual error brain	0.404	0.382	0.44	0.387	0.421	0.571	0.016	0.407	0.454	0.295	0.27
	(5.1)	(4.61)	(5.3)	(6.25)	(4.18)	(14.4)	(3.93)	(5.72)	(6.32)	(−)	(−)
Covariates											
Effect of pilocarpine induced SE											
Eff_SE_^c^	1.84	1.84	1.88	1.73	1.86	2.21	1.77	0.924	2.3	(−)	(−)
	(10.4)	(11.7)	(13.8)	(13.5)	(13.2)	(24.7)	(12.9)	(20.5)	(15.8)		
Effect of tariquidar											
Eff_tariquidar_ (3 mg/kg or 2 mg/kg)^d^	0.219	0.161	0.114	0.233	0.119	0.15	0.12	0.226	0.194	0.993	0.483
	(5.84)	(4.16)	(3.89)	(5.54)	(5.49)	(5.51)	(5.33)	(5.31)	(4.42)	(−)	(−)
Eff_tariquidar_ (15 mg/kg)^d^	0.168	0.135	0.101	0.215	0.101	0.134	0.103	0.148	0.155	(−)	(−)
	(5.22)	(6.94)	(7.71)	(6.56)	(9.3)	(7.61)	(9.01)	(8.65)	(7.74)	-	-
Effect of scan on tariquidar induced P-gp inhibition											
Eff_scan_^d^	(−)	1.33	1.29	1.37	1.29	1.25	1.21	1.52	1.4	(−)	2.28
	-	(5.67)	(6.78)	(5.47)	(7.1)	(6.21)	(6.08)	(6.97)	(6.06)	-	(−)

All (*R*)-[^11^C]verapamil plasma model parameter estimates outlined in Figure 2, obtained from final plasma model were fixed and the parameter estimate for the second brain compartment *V*_br2_ was fixed in rats to 2 ml. Parameters are given for the whole brain excluding (WB^*^) and including (WB) the increase of (*R*)-[^11^C]verapamil brain uptake during and after treatment with tariquidar, corpus striatum (CS), entorhinal cortex (EC), septal hippocampus (SHipp), temporal hippocampus (THipp), thalamus (TH), cerebellum (Cer), and frontal motor cortex (FMC) for rat; WB^*^ and WB for human. ^a^WB^*^ is the whole brain region excluding data from 60 to 140 min rat or 40 to 120 min human (baseline and post-inhibition scan are considered, as used for kinetic modeling). ^b^WB is the whole brain region including data from 60 to 140 min rat or 40 to 120 min human (baseline, during, and after tariquidar, and post-inhibition scan are considered). ^c^Eff_SE_ was a significant covariate on *V*_br1_. ^d^The effect of tariquidar, Eff_tariquidar_, is on *Q*_out_ the efflux clearance from the central brain compartment to the plasma compartment, while Eff_scan_ also takes into account changes occurring between scans (baseline or post-inhibition).

### Influence of tariquidar on (*R*)-[^11^C]verapamil transport across the BBB

To investigate the mechanism of P-gp inhibition, tariquidar treatment was included as a covariate for the influx clearance from the plasma compartment into the first brain compartment (*Q*_in_) or for the efflux clearance from the first brain compartment into the plasma compartment (*Q*_out_). The effect (*D*) of tariquidar treatment was best described as a combination of two categorical covariates and was defined as:

(5a) $$\begin{array}{c} \;D={{\text{Eff}}_{\text{tariquidar}3}}^{\left(\text{cov}\right)}*{{\text{Eff}}_{\text{scan}}}^{\left(\text{cov}\right)}\\ \;\text{cov}=0\;or\;1 \end{array}$$

(5b) $$\begin{array}{c} \;D={{\text{Eff}}_{\text{tariquidar}15}}^{\left(\text{cov}\right)}*{{\text{Eff}}_{\text{scan}}}^{\left(\text{cov}\right)}\\ \text{cov}=0\;or\;1 \end{array}$$

Eff_tariquidar3_ and Eff_tariquidar15_ are the estimated effects of tariquidar for the 3 and 15 mg/kg doses, respectively. The exponent, cov, was assigned to a value of 0 when no tariquidar was administered or 1 when tariquidar was administered. Eff_scan_ describes the difference in tariquidar effect between baseline and post-inhibition scans. The exponent, cov, of Eff_scan_ was assigned to a value of 0 and 1 for the baseline and post inhibition scan, respectively. The total effect of tariquidar inhibition, *D*, was therefore the fractional change in transport caused by tariquidar while also taking into account changes of tariquidar-induced P-gp inhibition occurring between the two scans (baseline or post-inhibition) as it is likely that the inhibition is changing due to the elimination of tariquidar from the plasma and brain.

**Figure 8:**
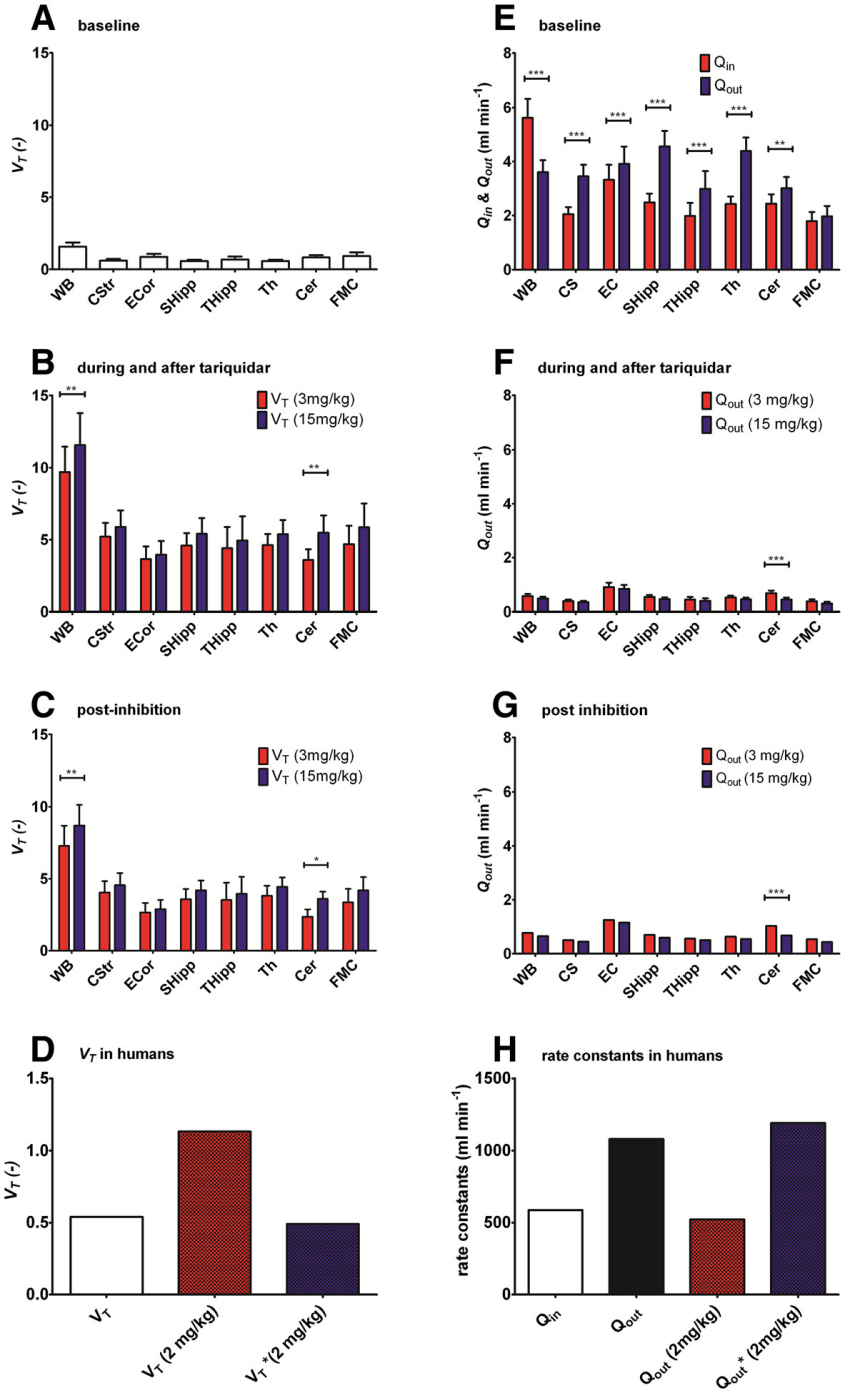
**Brain-to-plasma partition coefficients (A-D) and transport clearances across the BBB (E-H).** (**A**) *V*_T-NLME_ in rats at baseline, (**B**) *V*_T-NLME_ in rats during and after tariquidar administration, (**C**) *V*_T-NLME_ in rats in post-inhibition scan, and (**D**) *V*_T-NLME_ in WB in humans at baseline (*V*_T_), during and after tariquidar administration (*V*_T_ (2 mg/kg)) and in post-inhibition scan (V^*^_T_(2 mg/kg)). (**E**) *Q*_in_ and *Q*_out_ in rats at baseline, (**F**) *Q*_out_ in rats during and after tariquidar administration (3 or 15 mg/kg), (**G**) *Q*_out_ in rats in post-inhibition scan, and (**H**) *Q*_in_, *Q*_out_ at baseline, *Q*_out_ during and after tariquidar administration (2 mg/kg), and Q^*^_out_ in post-inhibition scan (2 mg/kg) in humans. *p < 0.05, **p < 0.01, ***p < 0.001; not significant *p* > 0.05.

OFV was found to be lower when tariquidar treatment affected *Q*_out_ (Equation 3) compared when tariquidar treatment affected *Q*_in_ (Equation 2) in all studied brain regions. For example, in WB, the effect of tariquidar on *Q*_out_ yielded an OFV (the lowest value corresponds to the best model) of −1,676, while the effect of tariquidar on *Q*_in_ resulted in an OFV of −1,541. Afterwards, the model was applied to the dataset, excluding data acquired during and after tariquidar administration. Again, the OFV was lower in all regions when the tariquidar effect was applied on *Q*_out_ compared to *Q*_in_. Parameter estimates, when including or excluding the data acquired during and immediately after tariquidar administration, were in general comparable. The parameter estimates for the whole brain regions when including (WB) and excluding (WB*) this data are given in Table 4. In Figure 8E, *Q*_in_ and *Q*_out_ values for baseline scans in rats are shown. At baseline, statistically significant differences between *Q*_in_ and *Q*_out_ were observed for all brain regions in rats, except for FMC. In all outlined brain regions *Q*_out_ was higher than *Q*_in_, while in WB, *Q*_in_ was higher than *Q*_out_. Additionally, in all brain regions, *Q*_out_ was decreased during and after tariquidar administration as compared with baseline scans (Figure 8F). Mean decrease in *Q*_out_ relative to baseline scan was −84±5% for 3 mg/kg and −86±4% for 15 mg/kg tariquidar. For the 3 mg/kg tariquidar group, largest decreases of *Q*_out_ relative to baseline scan were found for CS (−89%, Eff_tariquidar_ = 0.11) followed by SHipp (−88%, Eff_tariquidar_ = 0.12) and Th (−88%, Eff_tariquidar_ = 0.12), and smallest decreases were found for Cer and EC (both −77%, Eff_tariquidar_ = 0.23) (Table 4). For the 15 mg/kg tariquidar group, largest decreases in *Q*_out_ were found in CS, SHipp, and Th (all −90%, Eff_tariquidar_ = 0.10), and smallest decreases were found in EC (−79%, Eff_tariquidar_ = 0.215) (Table 4). In summary, there was a trend that *Q*_out_ was further reduced in 15 mg/kg as compared with 3 mg/kg tariquidar treated animals in all regions (Figure 8F). However, the difference in *Q*_out_ between 3 and the 15 mg/kg treated animals was statistically significant only in Cer. Also when comparing *Q*_out_ estimated when data during and immediately after tariquidar administration was excluded, Cer was the only region in which *Q*_out_ differed between 3 and the 15 mg/kg treated animals (Figure 8G). When excluding the data during and immediately after tariquidar administration, mean decrease of *Q*_out_ relative to baseline scans was −78±7% for 3 mg/kg and −82±6% for 15 mg/kg tariquidar (Figure 8G). Thus, *Q*_out_ was less decreased relative to baseline scans in the post-inhibition scan as compared to the last part of the first scan when tariquidar had been administered (comparison between Figure 8F and Figure 8G). In humans, *Q*_out_ was 1.8-fold higher than *Q*_in_ in the baseline scan (Figure 8D). *Q*_out_ during and after tariquidar administration was decreased by −52% compared with *Q*_out_ at baseline (Eff_tariquidar_ = 0.48) (Figure 8H, Table 4). During the post-inhibition scan, *Q*_out_ was completely restored to its baseline value (Eff_tariquidar_ = 0.99).

### Volume of distribution (*V*_T-NLME_)

*V*_T-NLME_ (*Q*_in_/*Q*_out_) values at baseline and after tariquidar administration are shown in Figure 8A,B,C,D for rats and humans. Tariquidar increased *V*_T-NLME_ by approximately 6.2-fold after 3 mg/kg dose and 7.4-fold after 15 mg/kg dose in the whole brain region when all data were analyzed (Figure 8C). *V*_T-NLME_ increase was lowest in EC (fourfold) and highest in CS (ninefold) for the 3 mg/kg tariquidar treated group. Also, for the 15 mg/kg tariquidar group, *V*_T-NLME_ increase was lowest in EC (fivefold) and highest in CS (tenfold). *V*_T-NLME_ was slightly decreased when the data acquired during and immediately after tariquidar administration (scan 1, 60 to 140 min) was excluded. This was the result of a slightly decreased Eff_tariquidar_ in the second scan compared to the first scan. In humans, a twofold increase in *V*_T-NLME_ relative to baseline was observed during and after tariquidar administration (Figure 8D). In contrast, *V*_T-NLME_ was unchanged in the post-inhibition scan as compared with baseline (Figure 8D).

### The influence of rat group (status epilepticus)

The model was improved when Eff_SE_ was included as a covariate for the volume of distribution of the central brain compartment *V*_br1_:

(6) $$V_{\text{br}1,\text{ind}}=V_{\text{br}1,\text{pop}}*{{\text{Eff}}_{\text{SE}}}^{\left(\text{cov}\right)}\;\text{cov}=0\;or\;1\text{,}$$

where Eff_SE_ is the estimated influence of pilocarpine-induced SE on *V*_br1_ (Equation 6) and implies a fractional difference between control and 48-h post SE rats. Hence, the exponent, cov, in Equation 6 was assigned to a value of 0 for the control rat group, while it was assigned to a value of 1 for the 48-h post SE group.

Eff_SE_ was found to be a significant covariate that increased *V*_br1_ in 48-h post SE rats as compared with control rats (see Figure 2, Table 4) in all brain regions except Cer. The largest increase in *V*_br1_ in the 48-h post SE group was observed for FMC (+130%, Eff_SE_ = 2.3) and in THipp (+121%, Eff_SE_= 2.21) (Table 4). In Cer, a small decrease in *V*_br1_ for 48-h post SE rats as compared with naïve rats was found (−8%, Eff_SE_ = 0.92). Eff_SE_ was not considered for the analysis of the human data set due to the fact that only healthy subjects participated in the clinical study.

## Discussion

In the present study, we used NLME modeling to study the PK of (*R*)-[^11^C]verapamil in plasma and brain, and the influence of tariquidar on (*R*)-[^11^C]verapamil brain PK to gain better insight into the mechanism of P-gp modulation by tariquidar. Moreover, our goal was to evaluate if the increase in brain activity induced by tariquidar during the first PET scan is better suited to describe regional and species differences and differences between control and post SE rats in cerebral P-gp function/expression as compared with using data from the post-inhibition scan alone. The developed model described the rat data well (Figure 6) and was then used to model the clinical data set. The small number of human subjects made it difficult to obtain estimates of the inter-individual variation in the human data set, but the model converged and, although the fit was not perfect, provided estimates of all structural model parameters. However, the variation in the human PK data set between the five subjects was rather large, and in combination with the few subjects, the results should be interpreted with some caution.

During model development we tested certain indirect response models [39] as used by Syvänen et al. [25], which either incorporate tariquidar plasma concentration or administered dose to describe the effect of tariquidar on (*R*)–^11^C]verapamil exchange between plasma and brain. These models described (*R*)–^11^C]verapamil kinetics well, but were not able to estimate the half maximum effect dose (ED_50_) of tariquidar for P-gp inhibition properly as our study only contained two dose groups (3 and 15 mg/kg tariquidar). Thus, a simplified model describing the effect of tariquidar as a categorical covariate was used. The decline in effect of tariquidar between the two scans, due to tariquidar elimination, was accounted for by a second categorical covariate (Eff_scan_). The appropriateness of the model was confirmed as Eff_tariquidar_*Eff_scan_ when including all data equaled Eff_tariquidar_ when omitting data during and immediately after tariquidar administration. We defined the NLME model in terms of clearances (ml/min) between model compartments and volumes of distribution (ml) as this is standard in conventional PK analysis. To compare different modeling approaches, we calculated *V*_T_ values from the outcome parameters obtained with NLME modeling (*V*_T-NLME_ as the ratio *Q*_in_/*Q*_out_) and compared them with the respective *V*_T-2T4K_ values obtained with standard PK modeling [9,30]. Overall, *V*_T-2T4K_ obtained with PET PK modeling and *V*_T-NLME_ obtained with NLME modeling were comparable. Tariquidar decreased the parameter *Q*_out_ which led to an increase in *V*_T-NLME_. This is in line with previous publications using standard PET PK modeling approaches, although the extent of the increase is somewhat larger in the present study as data during and immediately after the tariquidar administration were included (Table 5). This shows that excluding data during and immediately after P-gp inhibition leads to an underestimation of the effect of P-gp inhibition on brain concentrations of P-gp substrates. After P-gp inhibition in humans, *V*_T-2T4K_ was slightly increased, while a decrease was observed for *V*_T-NLME_. This is likely due to the fact that, in the standard PET approach, one subject is driving the average, while with NLME modeling, the model is less sensitive to one subject as all data are analyzed simultaneously.

**Table 5:** **Comparison of volumes of distribution (*V***_**T**_**) values of naïve, 48-h SE rats, and humans obtained with Logan analysis, PK modeling, and nonlinear mixed effects modeling, respectively**

**Sample**	**Logan analysis**^**a**^		**PK modeling**^**a**^**(2T4K)**		**Nonlinear mixed effects modeling**		
	WB*	**Cer**	WB*	**Cer**	WB*	**WB**	**Cer**
*Control rats*							
Baseline	1.6 (16)	2.0 (19)	1.8 (16)	2.2 (18)	1.5 (18)	1.6 (18)	0.81 (20)
Post-inhibition	7.6 (13)	6.1 (9)	7.8 (14)	6.1 (9)	7.0 (19)	9.6 (18)	2.4 (21)
Increase after 3 mg/kg tariquidar^b^	4.8-fold	3.1-fold	4.4-fold	2.8-fold	4.6-fold	6.2-fold	4.4-fold
*48-h post SE rats*							
Baseline	1.4 (8)	1.3 (7)	1.5 (12)	1.5 (6)	1.5	1.6	0.81
Post-inhibition	7.4 (11)	3.5 (13)	7.6 (12)	3.6 (14)	7.0	9.6	2.4
Increase after 3 mg/kg tariquidar^b^	5.5-fold	2.6-fold	5.1-fold	2.4-fold	4.6-fold	6.2-fold	4.4-fold
*Human*							
Baseline scan	0.64 (1)		0.65 (6)	-	0.54 (−)	0.51 (−)	-
Post-inhibition	0.79 (1)		0.80 (2)	-	0.49 (−)	0.79 (−)	-
Increase after 2 mg/kg tariquidar^b^	1.2-fold		1.2-fold	-	No difference	1.5-fold	-

*V*_T_ values were expressed as average and relative standard errors (%) for the WB region and Cer at baseline and post-inhibition scan. ^a^Logan and 2T4K results were obtained from Bankstahl et al. [9] for rats and from Wagner et al. [30] for humans. ^b^Increase after tariquidar administration for NLME modeling is based on the estimated covariate effect Eff_tariquidar_(1/Eff_tariquidar_).

The major findings of this study are as follows: First, it has been debated whether inhibition of P-gp affects the transport of substrates across the BBB into the brain (*K*_1_, *Q*_in_) or the transport out of the brain (*k*_2_, *Q*_out_). Two models have been suggested, i.e., influx hindrance and efflux enhancement. Influx hindrance can be described by the ‘gatekeeper’ model, where substrates are transported back from the lipid layers of the luminal cell membrane into the blood before they reach the cytoplasm. Efflux enhancement can be described by the ‘vacuum cleaner’ model [40] which suggests that substrates can be transported from the endothelial cells or brain parenchyma back into the blood. Thus, the question remains if tariquidar enhances brain distribution of (*R*)-^11^C]verapamil by increasing the influx (*K*_1_, *Q*_in_) or decreasing the efflux (*k*_2_, *Q*_out_) of the tracer. Our model clearly indicated that tariquidar enhances brain uptake of (*R*)-^11^C]verapamil by decreasing *Q*_out_ of the radiotracer. P-gp inhibition led to an on average sevenfold reduction in *Q*_out_ in rats (3 mg/kg tariquidar), while in humans (2 mg/kg tariquidar) a twofold reduction in *Q*_out_ was observed when all data were included into the model. When data during and immediately after tariquidar administration were excluded, an average fivefold reduction in *Q*_out_ was observed in rats. Thus, the reduction in *Q*_out_ when including the data obtained during and after tariquidar administration was more pronounced than when this part was excluded. This indicates that the effect of tariquidar on P-gp function is already declining at 2 h after tariquidar administration. In humans, the tariquidar effect on *Q*_out_ was apparent only during and after tariquidar administration and had completely disappeared in the post-inhibition scan. This highlights the importance of designing appropriate study protocols when investigating active transporters at the BBB as the onset and decline of inhibition is very rapid. Also, important to point out is that the analysis showed that *Q*_out_ and *Q*_in_ estimates were very similar regardless of whether the data from the tariquidar administration period were included or not, i.e., this confirms that the model is reliable and that only parameters that are expected to vary over time, e.g. the effect of tariquidar, are indeed changing.

Second, the study also indicated some regional and species differences in P-gp inhibition; large tariquidar-induced decreases in *Q*_out_ (CS, Th, and Hipp) indicated strongly enhanced brain uptake of (*R*)-^11^C]verapamil, while small decreases in *Q*_out_ (Cer, EC) indicated a weak enhancement in brain uptake of (*R*)-^11^C]verapamil as compared with baseline scans (Figure 8). A decrease in *Q*_out_ leads to an increased *V*_T-NLME_ as *V*_T-NLME_ is defined as the ratio between *Q*_in_ and *Q*_out_. Thus, the developed NLME model indicated that, after P-gp inhibition, *V*_T-NLME_ was significantly increased both in naïve and 48-h post SE rats. Inhibition with 3 mg/kg tariquidar resulted in regionally different enhancement of brain activity distribution, with weakest enhancement (low *V*_T-NLME_) in Cer and strongest enhancement (high *V*_T-NLME_) in CS and Th, similar to the findings of Kuntner et al. [10], who reported lowest *V*_T-2T4K_ increases in Cer and highest *V*_T-2T4K_ increases in Th of naïve rats after administration of 3 mg/kg tariquidar. (*R*)-^11^C]verapamil WB *V*_T-NLME_ was about threefold lower at baseline in humans than in rats (0.51 vs. 1.6±0.3) (Table 5). This is also in good agreement with findings from Bauer et al. [18] reporting twofold lower *V*_T-2T4K_ values in humans than in rats (Table 5). These observed differences could be due to different expression and transport capacity of P-gp.

Third, SE (Eff_SE_) was found to increase *V*_br1_ in most regions leading to an increase in brain exposure time of (*R*)-^11^C]verapamil in 48-h post SE rats compared with controls. This is mainly because an increase in *V*_br1_ indicates increased distribution of (*R*)-^11^C]verapamil to the slow equilibrating brain compartment (*V*_br2_). This in turn will slow down the elimination of (*R*)-^11^C]verapamil from the brain. The difference between the two groups was largest in FMC (Eff_SE_ = 2.3, Table 4). This is in line with results reported by Syvänen et al. [26], which showed that *V*_br1_ in WB was increased 1.3-fold in kainate-induced post SE rats. In contrast to all other regions, exposure time was decreased in Cer in 48-h post SE rats compared with controls (Eff_SE_ = 0.924, Table 4). In line with these findings, Bankstahl et al. reported an increase of *V*_T-2T4K_ in FMC, while Cer showed the largest decrease of *V*_T-2T4K_ in 48-h post SE rats compared with controls [9]. The decrease in *V*_T-2T4K_ in Cer of 48-h post SE rats was presumably caused by a twofold upregulation of P-gp as compared with control rats as revealed by post-mortem immunohistochemical analysis of the brain tissue [9]. Opposite to the findings presented in this paper, Bankstahl et al. also reported decreases or non-significant differences between controls and 48-h post SE rats in CS, Hipp, and Th. However, when ranking the regional SE-induced differences reported in the present study and by Bankstahl et al., the order is the same: FMC > CS > Hipp > Th > Cer. Bankstahl et al. found these regional differences only after partial inhibition of P-gp with 3 mg/kg. In the present study, all data were analyzed simultaneously including data from rats administered with 3 and 15 mg/kg tariquidar. This may, at least in part, be a reason for the differences in magnitude of regional differences between the present study and the study by Bankstahl et al. The present study showed that the effect of SE was mainly influencing the distribution of (*R*)-^11^C]verapamil within the brain (*V*_br1_) and not the actual transport across the BBB (*Q*_in_, *Q*_out_). It was possible to make this distinction by parameterization of the model using distribution volumes (the pharmacological term) and clearances instead of rate constants which depend on distribution volumes and clearances. Again, the effect of SE was the same when including and excluding data from the tariquidar administration period which indicated that the model parameter estimates are robust and do not change when adding or deleting some of the data set.

## Conclusion

This study showed that tariquidar enhances brain uptake of (*R*)-[^11^C]verapamil by decreasing the outflux (*Q*_out_) of the tracer across the BBB. Pilocarpine-induced SE did not directly influence (*R*)-[^11^C]verapamil transport across the BBB but had an indirect influence on the (*R*)-[^11^C]verapamil exposure time in brain by influencing the pharmacological volume of distribution in the brain (*V*_br1_). For the quantitative analysis of PET data, the NLME modeling approach used in this study is an interesting supplemental tool to standard PET PK modeling approaches on individual level to increase mechanistic knowledge of radiotracer transport across the BBB.

## Abbreviations

2T4K: Two tissue compartment model; BBB: Blood–brain barrier; Cer: Cerebellum region; CL: Systemic clearance; CS: Corpus striatum region; EC: Entorhinal cortex region; Eff_scan_: Effect of scan 2 as a fractional change from scan 1; Eff_SE_: Effect of pilocarpine-induced SE as a fractional change from control; Eff_tariquidar_: Effect of tariquidar treatment as a fractional change from no treatment; FMC: Frontal motor cortex region; NLME: Nonlinear mixed effects; OFV: Objective function value; PD: Pharmacodynamics; PET: Positron emission tomography; P-gp: P-glycoprotein; PK: Pharmacokinetics; *Q*_1_: Clearance from central compartment to first peripheral compartment; *Q*_2_: Clearance from central compartment to second peripheral compartment; *Q*_in_: Clearance from plasma to brain; *Q*_out_: Clearance from brain to plasma; SE: Status epilepticus; Shipp: Septal hippocampus region; SUV: Standardized uptake value; Th: Thalamus region; THipp: Temporal hippocampus region; *V*_br1_: Pharmacological distribution volume in first brain compartment; *V*_br2_: Pharmacological distribution volume in second brain compartment; *V*_c_: Pharmacological distribution volume in central compartment; *V*_p1_: Pharmacological distribution volume in first peripheral compartment; *V*_p2_: Pharmacological distribution volume in second peripheral compartment; *V*_T-Logan_: The brain-to-plasma partition coefficient obtained with Logan analysis; *V*_T-NLME_: The brain-to-plasma partition coefficient obtained with NLME; *V*_T-2T4K_: The brain-to-plasma partition coefficient obtained with 2T4K; WB: Whole brain region.

## Competing interests

The authors declare that they have no competing interests.

## Authors’ contributions

JM is the main author of the manuscript and performed all NLME modeling. CK performed the 2T4K modeling of the preclinical data and contributed to the interpretation of data. MB performed the human study including data acquisition and modeling. JPB contributed to the conception and design of the study, and acquired the preclinical data. MM and RAV contributed to the interpretation of data and drafting of the manuscript. OL contributed to the conception and design of the study, and was also involved in drafting the manuscript. SS contributed to the development of the NLME model and to the drafting of the manuscript. All authors read and approved the final manuscript.

## Authors’ information

JM is a post-graduate student developing new methods for analyzing PET data. CK, SS, and JPB are senior researchers focusing on preclinical and translational PET including pharmacokinetic modeling. MB is a senior researcher focusing on clinical PET. Professor RAV is an expert on animal models in epilepsy research. Professor MM is an expert in clinical pharmacology, and Professor OL specializes in the development of radiotracers for the imaging of CNS targets.
